# Practical Remarks on Blood-Letting

**Published:** 1866-06

**Authors:** D. B. Trimble

**Affiliations:** Chicago, Ill.


### CHICAGO MEDICAL EXAMINER.

N. S. DAVIS, M.D., Editor.

VOL. VII. JUNE, 1866. NO. 6.

### Original Contributions.

### ARTICLE XIX.

### PRACTICAL REMARKS ON BLOOD-LETTING.

[CONTINUED.]

By D. B. TRIMBLE, M.D., Chicago, Ill.

In many of the diseases affecting the Respiratory Organs, especially the Phlegmasiæ, blood-letting is indicated.

In *Pneumonia*, particularly the common acute variety, bleeding is generally required. I know that this has been a question in which great difference of opinion has prevailed within the few last years, the advocates and opponents of the measure being, probably, nearly equal in number; but I am fully convinced of its beneficial effect in many cases, and that in some cases it cannot safely be dispensed with.

In the early stages of the disease, where congestion of the lungs, to a greater or less extent is almost invariably an attendant, if the patient is of an ordinarily robust constitution, free venesection will, in many, perhaps nearly all cases, prevent the formation of the stage of hepatization. Nor should we, in this disease, be altogether guided by the condition of the pulse. If it should be full, frequent, and tense we need not be afraid to bleed freely; but there are some cases, in which the pulse is small and oppressed, which calls for this measure even more imperiously than where the pulse is in the first condition mentioned. This may be caused by the congestion of the lungs being so great as to prevent the free circulation of the blood through them. If the dyspnœa is severe, and there is a livid and turgid appearance of the face, with the contracted pulse, the opening of a vein will generally relieve the distress in breathing, and the pulse will rise with the flow of blood, in which event we may proceed with boldness; but if the pulse fails under the effect of the operation, we should close the vein. When the disease is not broken up by the first bleeding, it may be repeated until the force of the circulation, the pain and the dyspnœa are wholly or partially subdued, and where general bleeding is no longer admissible, local bleeding from the chest may be substituted. Bilious Pneumonia is sometimes a complication of inflammation of the lungs and liver conjointly, and in this case, the treatment requires no modification from that applicable to the preceding variety; but where it arises from malarial influences, venesection must be more cautious and sparingly practiced. In Typhoid Pneumonia the system is generally so prostrate as not to bear it at all, and in the chronic form it is seldom necessary or beneficial, except in some instances, locally. In the cases of children, also, if used at all, it should be locally.

Since writing the foregoing remarks, I have read a well written address by Dr. YOUNG, of Aurora, published in the February number of the *Examiner*. While concurring with his views of the pathological condition of the lungs in pneumonia and of its symptoms, and also the indications for treatment, I cannot arrive at the same deductions in regard to the effect of blood-letting. He remarks that, "In pneumonia we first have an intense injection of the capilliaries of the lungs. The blood is dammed up, and the heart's action increased in consequence. The substances of the lung, the cells and parenchyma are gorged with blood or bloody scrum. The functions of respiration and circulation are impeded, accelerated, and otherwise changed from their normal standard. There is too much blood, and not air enough in the lung, without any actual disorganization." "There is intense congestion of the lung, which constitutes the first stage of the disease—the period of high febrile action. If this engorged and congested condition be allowed to continue, the lung, after a time undergoes further alterations," etc. "The object to be accomplished in this stage, is to unload the lung and its capillaries of the surplus blood; to equalize the circulation, and lessen the heart's action," etc. Now, as far as these quotations go, I entirely agree with the writer, but when we come to the *means* of accomplishing this desirable object, I differ, and believe Dr. MILLARD'S view is the correct one. I know of no drug, not even veratrum viride, (the article advocated by Dr. YOUNG,) that can act so promptly or thoroughly in unloading the lungs of their surplus blood as venesection, and my experience teaches me that patients recover sooner "from the disease" *and* "the bleeding" than from the disease without the bleeding. Of course, in "anæmic cases," much more caution would be necessary in the use of this remedy.

I am at the present time attending a lady suffering from a severe attack of pneumonia. She is 24 years of age, florid complexion, sanguine temperament. I found her with a flushed countenance and congested eye, a full, bounding, tense pulse, severe headache, severe burning pain in the right lung, especially the upper portion, considerable dyspnœa, and some cough, but little expectoration. What was the indication? Certainly to unload the congested lung, and, as a sequence, the other symptoms. I opened a vein by a large orifice, and abstracted 12 or 14 ounces of blood, when she became sick and faint, and I stopped the bleeding. Presently she vomited freely; the pores of the skin were opened; she breathed freely; and said the pain in her chest and head had disappeared. I ordered warm emollient applications to the chest, and a cathartic to be given at daylight, (it was then about 9 o'clock P.M.,) and left her. The next day, I found the fever had returned, together with the pains in the head and chest, though not so severely; the dyspnœa was considerably less, and there was a free expectoration of viscid and rusty sputa. I gave neutral mixture with spts. nitre, and subsequently, added a small portion of tart. antim. On Saturday, the pain in the chest continuing severe, the pulse still full and frequent, the face flushed, and skin hot,

I gave her tinc. verat. viride, in 5 drop doses, every fourth hour. Three doses brought on active emesis, which I found much difficulty in allaying. I then applied a large blister to the right thoracic region, which produced thorough vesication, and relieved the pain. I also gave ½ gr. opii and 2 grs. calomel at night. On Monday, (yesterday,) I found her free of pain, free of fever, with a natural countenance, expectoration free, and nearly clear of the rusty appearance. In the evening, more fever, which I attributed partly to the sufferings produced by severe strangury. This morning, (April 3d,) there is some improvement; and I recommenced the veratrum, in doses of 2 drops, alternating it with an expectorant and diaphoretic. At my evening visit, I found the gastric distress renewed, with some emesis, and I discontinued the veratrum. *4th.* Gave expectorants and diaphoretics in alternation. *5th.* Fever nearly gone; pain in the lung disappeared; cough and expectoration free; continued expectorants and gentle anodynes.

This has not been a case to fairly test the effect of the veratrum vir., as it was preceded by venesection; but its failure to reduce the pulse, after the evidence of its influence on the system, as shown by the prompt and severe effect it had on the stomach, would certainly induce me to hesitate before substituting it for a remedy so long and so favorably known in the cure of this disease. There may, however, be cases, probably those occurring in anæmic or debilitated constitutions, where it might have a better effect than in the one in which I tried it; and from the evidence in its favor, by gentlemen prominent in the profession, it must, in many cases, exert a salutary influence; but while some of the latest and best authorities still advocate judicious bleeding in pneumonia, I do not think we are justified in ignoring it. Among these authorities I may cite Professor Jackson, of Boston, and Professor Walshe, of London, who says, "Pneumonia is an inflammation; hence antiphlogistic remedies, and among these blood-letting, must hold the first rank among means calculated to bring it to a favorable issue." "We find some men ascribing results the most favorable to venesection, pushed to startling extremes, and others tracing an equal or yet greater success to a system of absolute non-abstraction of blood. Lamentable, indeed, is the spectacle of hostile opinion and vacilating practice afforded by the history of the treatment of pneumonia, from the days of HIPPOCRATES to our own." Again, he says, "In acute sthenic pneumonia, here are few barriers to venesection." I fully concur in the conclusion he arrives at, that "We have learned from our predecessors the evils of *over-bleeding*,—and seem, in my opinion, very much disposed, at the present day, to learn from ourselves the evils of *under-bleeding*."

In *Acute Pleuritis*, free venesection affords almost immediate relief to the severe pain and dyspnœa that generally accompanies this disease. I have had patients who, from the severity of these symptoms, were unable to lie in the recumbent posture, and in whom respiration was performed with great difficulty, so greatly relieved by this means, that they would be enabled, before the bleeding was checked, to take a deep inspiration, and lie in any position. There is no disease in which such speedy relief is obtained from bleeding, perhaps, as in pleurisy, and none in which it is more necessary, except apoplexy. The operation my be repeated if the pulse remains strong and frequent, and the pain returns. I seldom find it requisite, however, to resort to more than one free bleeding. After the reduction of the most active symptoms, if the inflammation has not been subdued, cups or leeches applied to the chest will probably be required. There are often cases of pleurisy of so mild a character, as to yield readily to remedial means, without either general or local bleeding; and I have frequently treated cases successfully without resorting to either. Early in my practice, when what was then called congestive fever was prevailing, to a great extent, in my locality, I was attacked with pleurisy, quite severely, and being anxious to resume my professional duties as soon as possible, I took a free bleeding from the arm, applied a large blister to the chest, took no medicine internally, and in four days, was attending my patients.

In the combination of these two diseases, or *Pleura-Pneumonia*, it is as applicable as in either of the simple diseases.

In *Pneumo-thorax*, when inflammation of the pleura supervenes, leeching, and if the system will bear it, occasionally venesection may be resorted to.

In *Emphysema*, when there is congestion of the lungs, or bronchial inflammation, venesection, or local bleeding, either from the chest or between the scapulæ may be required.

In *Phthisis Pulmonalis*, the only circumstances that demand bleeding are a tendency to pneumonia, or hæmoptysis, and this only if the pulse is full and strong. In doubtful cases, it may be dispensed with, and only used locally by cups or leeches to the chest.

In inflammations of the *Respiratory Passages*, bleeding is often of essential benefit.

In *Acute Bronchitis*, though not required to the extent or as frequently as in pleuritis, (and, as a general rule, the mucous membranes require this treatment much less actively than the serous,) yet when the pulse is strong and excited, the dyspnœa severe, and there is much soreness of the chest, venesection may be resorted to in proportion to the strength of the patient, and the severity of the disease. Prof. WALSHE'S rule is: "In the first stage," where the disease is extensive, the constitution strong, fever of the sthenic type, "from 8 to 14 ounces may be drawn;" but should not be repeated unless the "blood drawn is highly hyperinotic." If the disease is moderate, or if violent in feeble persons, "cupping between the scapulæ, or 8 to 15 leeches to sternum infra-clavicular or axillary regions."

In the *chr nic* form, where there is much irritation, moderate general bleeding may, in some instances, though rarely, be necessary; local depletion will be more applicable.

In *Influenza*, it is seldom required.

In *Cynanche Treachealis*, of the catarrhal variety, the disease may often be relieved by other remedial measures; but if this is not the case, and there should remain considerable spasmodic action, with a full and vigorous pulse, blood should be taken from the arm; and, perhaps, in a few very urgent cases, the temporal artery or jugular vein may be opened. Bleeding in children, however, should never be carried to syncope. If fever, difficult respiration, pain within the throat or chest, and hoarseness of voice remain, evincing the continuation of inflammation, leeches applied to the throat or chest will often afford great relief. In *Pseudo-Membranous* croup, bleeding is seldom required; and in those cases following scarlatina, should never be used. In cases from other causes, when the inflammation is severe, leeching the throat or chest, or moderate venesection, may sometimes be useful.

In *Acute Laryngitis*, if the fever is high, with a full, strong pulse, free venesection, repeated if necessary, and followed by leeches applied over the larynx, if the disease is not previously subdued, will be frequently required. In *Chronic Laryngitis*, general bleeding is scarcely ever requisite; but leeches to the larynx, or cups to the nuchal region, are often useful.

*Coryza*, accompanied by headache, and a full, strong pulse, may be much relieved by moderate venesection; and if the frontal or maxillary sinuses are inflamed, leeches should be applied to the forehead, cheeks, or nostrils.

In *Pertussis*, accompanied by severe bronchial inflammation, venesection or leeching the chest may be resorted to; but in the great majority of cases, it is not demanded.

During the paroxysm in *Asthma*, if the pulse is full and strong, and there are symptoms of accompanying bronchitis, venesection will often give prompt and efficient relief. In the interval, if there is chronic bronchial inflammation, local depletion from the chest will be appropriate.

In inflammation of the *Bronchial Glands*, local depletion may be beneficial, if the disease is not produced by tuberculous or malignant causes.

Where congestion of the brain is present from *Apnœa*, venesection is sometimes necessary; when it should be taken from the jugular vein, as this relieves the brain more directly, and also the right side of the heart, which is usually overloaded.

